# May I long experience the joy of healing: professional and personal wellbeing among physicians from a Canadian province

**DOI:** 10.1186/1471-2296-10-18

**Published:** 2009-02-24

**Authors:** Brenda L Lovell, Raymond T Lee, Erica Frank

**Affiliations:** 1 University of Manitoba, Department of Business Administration, 181 Freedman Crescent, Winnipeg, Manitoba, Canada; 2 University of British Columbia, School of Population and Public Health, 5804 Fairview Avenue, Vancouver, British Columbia, Canada

## Abstract

**Background:**

The development of best practices to promote physician wellbeing at the individual and organisational levels is receiving increased attention. Few studies have documented how physicians perceive their wellbeing in these contexts. The purpose of this qualitative study is to identify and discuss the reported factors that hinder wellbeing, as well as the reported factors that would promote wellbeing among physicians.

**Methods:**

There were 165 physicians from a province of Canada who wrote their open-ended responses to two questions, as part of a larger self-report questionnaire. The questions asked what causes them stress, and what interventions should be implemented at organisational/institutional levels. The largest specialty was family medicine, followed by internal medicine, and surgical disciplines, with 58% of participants male. A general inductive approach was used to analyze the data and themes and sub-themes were discovered using the socio-ecological model as the framework.

**Results:**

Reponses were both personal and professional which resulted in the emergence of four major themes to reflect this diversity. These themes were external constraints on the practice of medicine, issues at the professional/institutional levels, issues at the individual practice level, and work/life balance. The work/life balance theme received the highest number of responses followed by external constraints on the practice of medicine. In the major theme of work-life balance, work-life conflict received the most responses, and in the major theme of external constraints on practice of medicine, lack of resources (human and material) and restrictions to autonomy received the most responses. Ideas for interventions in the work/life balance theme were health promotion, and healthy workplace initiatives. In the second largest theme, suggested ideas for interventions were collegiality/professionalism and policy formulation at the health care system.

**Conclusion:**

Our findings have implications for governance and health policy, health human resources and education. In particular, the socio-ecological framework was a useful framework to analyse physician wellbeing due to its applicability for issues at the structural, organisational, and individual levels. Future research should target interventions at the organisational and institutional levels to address work-life conflict and job dissatisfaction.

## Background

"In seeking absolute truth we aim at the unattainable and must be content with broken portions."

Sir William Osler, Canadian physician

The World Health Organisation (WHO) has declared that an understanding of health should be deeply rooted in human nature and societal structures. They define health, synonymous with wellbeing, as "a state of complete physical, mental, and social wellbeing and not merely the absence of disease." In a broader sense, this includes "the extent to which an individual or group is able to realise aspirations, satisfy needs, and to change or cope with the environment."[1]

Research on the health and wellbeing of physicians is increasing. Studies in the United States show excellent physical health and low mortality for physicians, typically even better than other individuals of high socioeconomic status. [2,3] In contrast, research from Norway indicates that physicians have high levels of subjective health complaints, especially among females. [4] In addition, other European research indicates that psychiatric morbidity and cardiovascular mortality rates among physicians are the same as or higher than other professional groups. [5] Complaints of excessive workload, high levels of stress and dissatisfaction, threaten to undermine motivation and health, and can result in poor performance and medical errors. [6-8] These studies have also demonstrated a direct link between health care workforce capacity and patient safety with indications that working conditions affect providers' abilities to provide quality care. [6-8] This is thought to occur in part due to a lack of effective management and supportive work environments, which undermine attempts to improve working practices. [5-8] Institutions that have better communication, greater emphasis on training, and staff autonomy reduce provider stress with better patient outcomes.[5,7,8] Research from the United States indicated that control over the practice environment, collegial support and adequate resources were associated with higher levels of satisfaction and organisational commitment.[9] An interventionist study done in Sweden indicated that organisational support improves work satisfaction and mental energy, and decreases work related exhaustion.[10] This points to the need for health care organisations to take steps to create a positive and supportive work environment. Sufficient numbers of physicians may not automatically result in improved patient care, unless there are good working conditions, coupled with comprehensive human resource strategies to motivate and retain physicians. [5,6]

The overall aim of our study was to determine the factors that contribute to and undermine physician wellbeing from a province of Canada. We chose to use a mixed method design to gain a more in-depth understanding of our overall aims, with information to inform our study drawn from the practice environment. [11,12] The objectives of our quantitative study were to test and evaluate relationships among predetermined variables using established scales and measures to determine the degree of work related stress and strain. The objectives of our qualitative study were to identify and discuss challenges, stressors and suggestions for interventions using open-ended questions. We used open-ended questions to allow for a greater range of responses. In this paper, we present our findings from the qualitative component of our study, drawing upon literature from Australia, New Zealand, Canada, United States, and Europe using MEDLINE and SCOPUS as the data bases. We will then discuss our findings and their implications for governance and health policy, health human resources and education. Finally, we will discuss the strengths and weaknesses of our study, compare with past research, and suggest directions for future research.

### Patients and patient care

Misinformed patients, high patient volumes, long wait times and delays, have a negative impact on the emotions of physicians and patients, which manifests in anger and frustration for both parties.[13] Patients and their families seek greater accountability, and if they feel that their concerns have not been adequately addressed, disgruntlement and formal complaints result. [14,15]Threats of retaliation via media or the formal disciplinary process by patients and families impact the decision making process of physicians, with family physicians reporting the highest negative experiences. [14] Research from Australia found that inadequate communication was the leading cause of complaints, and is indicative of the changing public expectations of the profession. [15]

The strength of the patient-physician relationship is highly associated with physicians' satisfaction, especially for family physicians. [16] Time restrictions, coupled with pressures to see larger volumes of patients, strain this relationship, and also present a barrier to the integration of the psychosocial aspects of medical care. [16] Potential for conflict and confrontation with patients is a cause of extreme anxiety for physicians, and may precipitate avoidance behaviours to curtail patient discontentment or reproach. This can result in unnecessary tests, treatments and prescriptions. [17]

### Medical practice

Continuing medical education (CME) is a post-graduate licensure requirement for all Canadian physicians. [18] The advent of continuing professional development (CPD) is suggested as a way to incorporate the social, political and cultural influences on patient care, as well as the learning needs of managerial physicians and directors.[18] These physicians experience especially high levels of stress as they must adopt a dual work perspective to be compatible with clinicians and institutional management. [18] This requires superior skills in areas such as personnel management, communication skills, negotiations, quality assurance, and organisational management, none of which are traditionally covered in medical education. [6,18] The practice environment is subject to myriad direct and indirect influences on medical practice, thus educational and research opportunities should be derived from an accurate understanding of this work environment. [18]

Finally, research into the ergonomic design and layout of medical work spaces helps prevent potential health hazards for physicians and patients.[5,19] German surgeons indicated that problems with lighting and positioning of monitors, difficulty in handling equipment and instruments, and hazardous cables and tubes all have the potential to create disturbances and physical impairment. The authors suggest that research in this area is needed to prevent hazards in the work environment, which will also improve patient safety. [19]

### Medical Culture

Students entering medical school experience long hours, sleep deprivation, and limited time for personal activities, as well as observing death and suffering. For international students, the burden is even greater, as they are adapting to a new cultural environment. [20,21] Physical illness, depression and other emotional problems can result from the stress and isolation, unless the appropriate coping mechanisms are utilised during medical school training.[21] These concepts should be incorporated into medical curricula early, as students who suffer from distress in the first year of training are at the highest risk of developing symptoms of stress later on. [21]

Students and junior doctors experience uncivil workplace behaviours such as bullying, belittlement, and racism creating a toxic environment. These acts have far reaching negative effects on personal health, personal relations, career satisfaction, and create a ripple effect that permeates every facet of the health system. Research on medical students, [22] residents, [23] junior doctors, [24] and physicians in practice, [25] suggests that this is a serious problem of multi-country and continuing scope and warrants further research and policy development. Healthy workplace initiatives are needed as residents and medical students indicate that medical training has adversely affected their health. [23,26]

Quality patient care requires good working relationships across disciplines and specialties. A lack of collegiality plays a central role in the fragmentation of care, patient dissatisfaction, poor outcomes and adverse affects on physician clinical performance. [7] In addition patient injury and medical error are highly stressful events, negatively impacting physicians professionally and personally. [27] Supportive collegial relationships can help to mitigate the effects of stressful work, improve retention, and promote quality and safety. [7,9,27] In particular rural and remote physicians face professional, cultural, social and geographical isolation and as a result, support networks should be incorporated into retention strategies in rural and remote locations. [6] Medical work is collaborative by its nature and is based on the sharing of experiences and knowledge between colleagues; the obligation to help ones colleagues and behave collegially is part of the Hippocratic Oath. The Finnish Medical Association has formulated a special code of medical collegiality which provides guidelines for behaviour according to principles of mutual respect among physicians. [28]

### Organisational Culture

Devolving health care planning and decision-making from provincial governments to regional health authorities in Canada were done to contain costs, improve health outcomes, and better integrate and coordinate services. [29]This granting of rights to reorganise and reform service delivery has created tension between governmental expectations, provider interests, and patients' needs and desires. [29] Woven within this tapestry are diverse professional sub-cultures whose values and practices may diverge from the values and practices of the organisational culture. Strategy-culture congruence results in superior patient care, whereas poor congruity causes conflicting interests and resistance to implementation. [29] Physicians, the dominant professional sub-culture, were excluded from boards, and subsequently felt left out from channels of advice and influence, at a time of federal health care cuts on their medical practices. [29]

Reports have indicated that important sources of physician satisfaction such as clinical autonomy, [9,30] have eroded with the introduction of managed care budgets, bureaucracy and guidelines. [5,16] Sources of satisfaction such as control over one's work protect against work-related stress, and are important determinants of health and wellbeing. [10,30] Tensions arise if explicit reliance on these performance evaluators are used for health care planning and decision making[5,6,29,31] without considering the practitioner's element of discretion, duty of non-malfeasance, the contextual complexities, uncertainty in medicine, and differing variations and needs of patients.[31]There is dissatisfaction in the doctor/manager relationship, which will dampen efforts to procure quality care. [31]

### Complaints and Disciplinary Process

Industrialised societies expect to receive an optimal standard of medical care. If patients perceive that suboptimal care has been provided, they may seek redress against physicians by registering a complaint. The complaint process should improve medical practice, and provide an acceptable outcome for patients, without accentuating blame and punishment. However, the typical effect on physicians from this process is considerable stress, guilt, depression, and reduced enjoyment in medical practice. [32] Research from New Zealand has indicated that complaints have potential to impact negatively on quality of care, damage trust and goodwill, and increase defensive medicine. [17,32]

### Health Human Resources

Many health systems have material shortages, and lack funds for capital expenditure. Likewise health worker shortages are global problems, especially in remote, less developed, and rural locations. [6] The World Health Report 2000 defines health human resources as "the stock of all individuals engaged in promoting, protecting or improving the health of populations."[6] In addition, internationally trained physicians who may have immigrated to Canada, endure the stress of acculturation, and the difficulties associated with gaining licensure.[20] Single female physicians are particularly vulnerable because of a lack of faculty role models, experiences of isolation, and sexism. [22,23,25]

An imbalance between personal and professional life causes work/life conflict, a major determinant of emotional exhaustion and stress for physicians due to heavy patient loads, long hours, and unpredictability. [8,33] The stress and strain of a medical career impacts life outside of work, resulting in difficulties maintaining personal engagements, family responsibilities, and health. Maintaining good physical and mental health, particularly for more emotionally demanding fields (e.g., oncology and critical care), and physically demanding fields (e.g., surgical disciplines) is paramount. German surgeons stand 95% of their time while operating and are often in positions that cause discomfort or pain, requiring pain relievers and physiotherapy. [4,19] Oncologists at the Mayo Clinic reported that patient loads, death and suffering were significant stressors, followed by balancing personal and professional life. [34] Key wellness strategies identified were the nurturance of personal and professional relationships, finding meaning in work, self care practices, and development of a life philosophy/spiritual practice. [34]

There are indications that high percentages of physicians and residents do not have family physicians, and are diagnosing, self-medicating, or prescribing for their own illnesses, despite recommendations that they not do so. [8,23,26] Mental health admissions were seen as being particularly troublesome by the physicians, as they feared recrimination and social stigma. [23,26]There is good news, however. The scientific literature supports intuition: health care providers preach what they practice. Analyses of physicians' physical health behaviours in the United States have shown that physicians and medical students have healthier personal habits than those of the general population, and that those who have the healthiest personal habits are more likely to encourage patients to adopt such habits.[2] These healthy behaviours, may not be universal, as a research study of Maltese doctors found that 63% rarely exercise, record high BMI, and have infrequent cholesterol checks, with potential negative effects for patients. [35]

## Methods

Self-report surveys were sent in alphabetical order by last name to registered physicians in the province of Manitoba, Canada during the fall of 2006 through the bi-monthly newsletter sent by the provincial medical association to its members, and a direct mail-out by the researchers to members of the provincial College of Physicians and Surgeons. Ethics approval was obtained from the University of Manitoba Health Research Ethics Board and responses were returned to the first author's university mailing address. We used a mixed method research design where the information is collected, analyzed, and mixed with inference from both qualitative and quantitative data in a program of inquiry. [11,12,36] This provides for the use of both varieties of data to inform efforts to plan interventions. [11]Of the five mixed method purposes we used expansion to aim for scope and breadth by using multiple methods for different inquiry components, which were kept separate. [12,36]

We received responses from 165 physicians who wrote their answers to the following two open-ended questions: "What are some of the factors that cause stress in your life that have not been covered in this survey?" and "What would you recommend to the provincial branch of the College of Physicians and Surgeons or the provincial medical association to help its members better cope with the challenges of their profession?" These open-ended questions were developed to gain insight into contextual problems as an expansion to the quantitative component of our study. [12,36] The two questions addressed "what" and "how" by asking the participants to provide additional information on stressors they encountered (what are the factors causing stress) and suggestions for interventions (how can these challenges be addressed). [12] The breakdown of the participants is provided in Table 1.

**Table 1:** Physician demographic characteristics

**Physician specialty**		**Physician gender**		**Practice location**		**Organisation**	
Family medicine	71	Male	96	Urban	132	Manitoba Medical Association	86

Internal medicine	39	Female	69	Rural	33	College of Physicians & Surgeons, Manitoba	79

Surgical disciplines	16						

Paediatric disciplines	12						

Residents	11						

Psychiatry	11						

Other	5						

The raw data were typed verbatim into Microsoft Excel, followed by in-depth reading and interpreting by the first author. A coding framework was developed which allowed four major themes to emerge, with some individual text fitting into one theme, and others into two or more different themes. These initial themes were influenced by our research questions with Bronfenbrenner's ecological systems theory used as the framework to create the major themes. [37] Founded on the premise that behaviour is a function of the person and environment, this framework has been used to study the interaction between the environment and human development in wide ranging applications. This approach describes factors from four different systems that operate and exert influence on individual behaviour at the macro, meso, exo, and micro levels. [37] Four themes were developed for this paper with reference to these levels which exert influence on physicians. Although the themes are each distinct, they are interactive and interdependent, and for these reasons ecological systems theory was used as the framework for our study. In keeping with this model, the macro level would be "external constraints on the practice of medicine", where we see how the health care system directly influences and regulates the health care working environment. The meso level are "issues at the institutional and professional levels" including organisations and institutions that shape work structure, relations, and rules/policies. Physicians at this level are required to be active participants with licensure, and continuing education as examples. The exo level would be "issues at the individual practice level" including professional and patient relationships, and organisational and community networks which affect the individual, with varying degrees of participation. The micro level consists of aspects of the environment within the immediate social and physical domains of the individual which affect psychological and cognitive factors such as personality, behaviours and beliefs. [37] A general inductive approach for analyzing the data to create the sub-themes was used. [38] The specific sub-theme categories were derived from multiple readings of the raw data until saturation occurred, then the marked text segments were copied into the emergent categories. [38] The major themes and sub-themes were analyzed for accuracy by the second author, an industrial psychologist with specialized knowledge in work and wellbeing, and the third author, a physician with specialized knowledge in the area of physician health and wellbeing. All authors agreed with the classifications and themes created by the first author, so no inter-rater statistics were required.

## Results

In this section we present the answers to the two open-ended questions asked of the physicians. Stressors and ideas for interventions have been identified at the individual, professional, and organisational levels. As suggested by Arnetz, [5] a holistic conceptual framework using a socio-ecological perspective is appropriate to incorporate the many stressors inherent in medicine and is reflected in our themes and sub-themes.

### Theme One: External Constraints on the Practice of Medicine (Macro level)

#### Stresses and Difficulties

##### Professional Autonomy

Autonomy is the power granted to physicians to have control over their own work, and to be responsible for overall patient care. Several of the surveyed physicians felt that their autonomy has been constrained and have elaborated as follows:

Feeling of loss of control due to numerous government guidelines & protocols.

Bureaucracy in workplace can't make any decisions for myself.

Consumerism vs. professional autonomy.

Bureaucratic interference in medicine.

##### Workplace Incivility

These are actions that create a hostile work climate through overt and covert behaviours such as belittlement, harassment, bullying, work overload and undermining, causing mental and physical distress, turnover, and reduced productivity. These behaviours hamper morale, quality of care and inhibit collaboration and are stated as follows:

Interactions with co-workers, rude/obnoxious co-workers, bullies.

Racism faced on the job (from colleagues).

Second guessing your-self to avoid unnecessary/inappropriate criticism by colleagues.

Judgmental environment (patients and colleagues)

##### Professionalism

Professionalism combines skills, attitudes, and behaviours of a collective group, which includes continuing education, integrity, ethical behaviour, honesty, respect for others, and adherence to professional codes. Lack of collegiality results in fragmented care, reduces job satisfaction, and several physicians desire better adherence to medicine's higher values. They express:

Worry about how others at work perceive your work.

Difficulty of cooperating fully with colleagues because of competitive aspects of the business side of medicine.

Lack of collegial support and lack of time to communicate.

Unprofessionalism and/or incompetence in medical/nursing colleagues.

Frankly I am very pessimistic medicine remains fraught with an exaggerated work ethic, it remains a macho culture.

Changing relationship between community doctors and hospitals/specialists.

##### Health Human Resource Challenges

Shortages exist for all types of health care disciplines, which creates difficulties to maintain services, constraining physicians in their medical practices. The effects on the physicians and their practices are far-reaching and this is of the greatest concern for physicians elaborated on as follows:

I think shortage of health care workers, leading to diminished time with clients, drives many of the difficulties in profession.

Chronic understaffing in the hospital setting, resulting in physician inability to implement treatment plans. We're not responsible for treatment delays, but are made to bear the brunt of the repercussions.

No accountability of administrative staff.

Emergency room on call conflicting with regular office hours. I gave up on call coverage 30 months ago.

##### Public Health Care Systems

Publicly funded health systems are facing escalating costs, government cutbacks and rationing, which creates difficulties for physicians to access needed resources and equipment. This has a detrimental effect on individual medical practice and the effects are described as follows:

The biggest stresses at work are related to the health care system. Inadequacies, bed shortages, waits for consults etc., combined with elevated expectations by patients, society etc.

Finding appropriate in-hospital resources to properly care for my patients.

Difficulty arranging specialists/tests for patients in a timely manner.

The length of wait times for diagnostic imaging/biopsies that delays definitive surgery.

Prolonged slowdowns to definitive surgery because of OR cut backs i.e. 10 weeks every summer.

Difficulty obtaining services and its impact on communications and stress for physician. Stress related to systems.

##### Health Care Administration

The governments' response to demands for universal access to health care created regional health authorities. These are responsible for planning and implementing delivery of health care services, which has created greater bureaucratisation. Physicians describe the impact of this on their medical practice:

Administrative decisions that come from higher up that add stress to my job.

Administration, bureaucrats, love my work/patients, system doing me in!

I am a psychiatrist and do not have hospital privileges so trying to get a really sick patient a hospital bed is stressful, and following that patient for at times weeks, because there aren't any beds is stressful. At times I have daily contact with really ill patients and often hours and hours on the telephone, cumulating over the course of 2–3 weeks with patient, family member etc. None of this is reimbursed, but does affect my life.

### Theme One: External Constraints on the Practice of Medicine

#### Ideas for Interventions

##### Professional Values

There is better need to develop values as a professional group in order to support better quality of care, and to enhance supportive networks and relationships. Physicians suggest the following as ways to encourage professionalism:

Emphasize the fun of the profession, outweighs by far ones income.

Programs to recognize and encourage inter-physician support is lacking. Could be ethically based network support building exercise.

The most helpful factors for me are having control over aspects of my work life and very supportive/fun colleagues.

Hippocratic Oath be stressed and followed i.e. my colleagues will be my brother (or sister), teach & be taught.

Ensure all members maintain high standards and not succumb to societal, political or unfounded consumer pressures.

Empower/teach physicians how to take back some of the control in the medical system.

##### Inter-professional Collaboration

The following physicians feel that working with other health care professionals as part of a collaborative team effort will enhance quality of care and improve medical practice:

A much more collaborative approach with health care professional colleagues, nurses, social workers, psychologists, counsellors, ethicists, communicators.

Increase support for other health care professionals as part of the primary care system.

Need to enhance the profile of the profession in terms of profile with other professions (pharmacists, nurses, administrators).

##### Health Human Resource Strategies

Finding and keeping physicians requires both intrinsic and extrinsic rewards. Health care is interdisciplinary, and shortages of other health providers across disciplines, places a strain on the entire system. Strategies for retention are provided as follows:

Support rural physicians with policies that enable easier recruitment & retention.

Higher pay is good, but good working conditions still most important – sometimes physicians don't recognize this.

Take a more assertive role in retaining skilled para-professional i.e. nurses.

##### Public Education

Using principles of adult learning and prevention, this involves addressing current issues in the public health realm. Much greater involvement in educating the public needs to occur in a collaborative fashion, and physicians reiterate as follows:

Help to educate the public and government to the reality that we're trained to a very high level but cannot supply care to the same elevated level when resources are not at the same level.

Get politicians to fund a campaign of information about the position of the physicians/family physicians in the health care system (re decision-making) for the general population, because physicians being in the firstline of health care are frequently blamed for shortcomings of the health care system.

##### Reducing Health Care Administration

The additional layers of paperwork and administration due to health care administration, makes it harder to cope with an already stressful and busy work schedule, and physicians recommend that to help them manage, the following should be done:

Stronger stance against administration and more resources where rubber meets the road. Its becoming harder to get the smallest things done.

There needs to be less bureaucracy and regulation by the authorities, ........ to stop "protecting their turf" and cooperate to help patients and doctors.

Better and stronger advocacy for accountability within the hospitals...... and entire health system. A cost per case (patient) would be a necessity.

Streamline licensing procedures and other paperwork that physicians must do.

Find a way to be paid for multiple problems in one visit i.e. be able to bill for the four or five problems patients bring in rather than one "diagnosis" per visit with payment for that one "diagnosis".

### Theme Two: Issues at the Professional/Institutional Levels (Meso level)

#### Stresses and Difficulties

##### Licensure and Patient Complaints Process

Physicians indicate that improvements are needed regarding how patient and family complaints and the licensure process are handled by the provincial medical regulatory body:

Patient complaints and how they are dealt with. It seems that doctors are put on the defensive with little or no support from any complaints – trivial or otherwise.

Unwarranted bias ....... concerning trivial patient complaints, encouraging patients to complain.

Do not threaten us make us feel like humans, and not sheep. Respect us as we do respect you. Make room for our real talents and skills and not treating us like a piece of paperwork. South Africans feel negative in general. Can we give them more to feel wanted?

##### Medical Training

Some physicians felt that that support around emotional, counselling and psychological needs should be stronger, especially during their training:

Inadequate university support for training physicians/residents.

I feel strongly that skills to "cope with the challenges of the profession" needs to be addressed at the medical student level more effectively.

Improve education of medical students to be more culturally aware.

Make better choices regarding specialty they choose.

##### Organisational/Professional Culture Clashes

Differences in values cause clashes between the dominant professional sub-culture and organisational culture and ultimately decrease quality of care and are described as follows:

The feeling that "big brother" is watching you, frustration at not having skills recognized by the authorities.

Pressure from "authority" to see more patients, work faster.

Getting treated as if I am a child, feeling as if every action is being monitored.

Intense scrutiny and unproductive criticism from bosses.

### Theme Two: Issues at the Professional/Institutional Levels

#### Ideas for Interventions

##### Improving Patient Complaints Process

To be confronted with a complaint is very stressful, and can cause severe disruption and threats to ones medical practice, and the following have been suggested to improve upon this process:

Be more supportive toward physicians especially when complaints arise. Less formal approach toward resolution of patient/family complaints and faster resolution.

Not to act as a punishing institution, but rather as an educational and a damage controlling agency/teaching agency.

Disregard/deal with more empathy with frivolous complaints from patients and colleagues. Deal more fairly with physicians needing help.

Provide info on conflict/mediation services proactively, so doctors can meet these people before difficulties arise and contact them early, not late during problems.

##### Physician Support Mechanisms

Medical practice is inherently stressful. Supportive mechanisms in an atmosphere of impartiality are beneficial to physical and mental health and help to prevent burnout:

Confidential physician help line for advice, support information etc.

Increased awareness of problem, de-stigmatize trouble coping.

Counselling services (not addiction related)

##### Reducing Organisational/Professional Cultural Clashes

Lack of mutual goals between managers and physicians creates adversity, and the following have been suggested as ways in which to bridge this gap:

Maybe have evening sessions every few months to discuss concerns etc. ...... ensuring that management has a clue what MD's life is like (e.g. seminar)

Better understand our frustrations.

### Theme Three: Issues at the Individual Practice Level (Exo level)

#### Stresses and Difficulties

##### Patients and Patient Care

Patient negative emotions are difficult to manage, and the frustration is evident in the written comments by three physicians:

We're not meant to be doormats. The increasingly and aggressive demanding attitude of patients.

Patients very confrontational.

Angry patients.

A retired physician has now assumed a different role as a patient and wrote the following comment:

In my years of active practice it would seem unnecessary to give particular thought to the many categories listed in the questionnaire. Probably because most physicians were dedicated to the practice full-time because of the positive response received from the patients. Financial matters were rarely discussed, the hours devoted to patient care were not recorded – patients were treated as people with medical problems, not as just medical problems. In other words, we cared for people the way we would want to be treated. I am aware of the gradual change in attitude of many practitioners.... Having required a lot of surgical medical care in the last six years, I am happy to benefit from the advances in surgery but not happy in the total (art) of patient care.

The proliferation of health information provided via the media is not always accurate. This influences patients' views of illness and decision-making, and concern is expressed by one physician:

Inappropriate internet and lay press information.

Physicians experience disappointment and misgivings because of mitigating factors that interfere with patient care and desired outcomes:

Patients make numerous excuses why they can't change behaviour as roadblocks to change (especially with regards to obesity). They are unwilling to accept that they need to do something to help themselves i.e. want a quick fix.

Patients whose needs cannot be met.

Inability to give best treatment due to inadequate facilities and staffing.

When treatments do not work as I had hoped.

The North American culture is more dangerous to the health of people than any other culture I know e.g. eating too much, eating the wrong things, spending too much time in front of TV or computer.

Lack of follow-through is present in all patients – born Canadians as much as more recent citizens. Patients' cultural beliefs do not interfere with my diagnosis at all – but I recognize that they often do not follow my recommended treatment – which is their privilege.

##### Practice Management

Maintenance of medical practice includes administration associated with patient care, and the following physician comments describe the stresses that form part of this function:

Worried about being audited, worried about doing something wrong, missing something important.

Extreme difficulty in getting as well as retaining competent office assistant – I have had a run of poor assistants for the past 18 months!

Keeping staff happy and ensuring enough staff.

One administrative physician discusses the difficulties associated with a managerial role

Management decisions.... I am middle mgmt.

High volumes of patients, interruptions, and miscommunication causes delays and frustration:

Double booked appointments and suddenly being asked to drop what I am doing to accommodate someone who didn't organize their time well.

A cumbersome or inadequate physical work space affects patient safety, concentration and health. A physician and surgeon describe problems with their physical work environment:

Poor organisation of operating room.

Loud environment, constantly being interrupted by RN's, other MD's, secretaries etc., no personal space, MD's work in one big room.

##### Medical Knowledge

Physicians are required to participate in post-graduate education activities to maintain licensure, and to keep abreast of new advances in medical care. Medicine continues to evolve and change with new procedures, protocols, and greater expectations, which causes stress to keep up:

Trying to keep up to date.

Concerns about lack of knowledge and expertise.

Fear of failure.

Being bombarded by literature and drug company info.

### Theme Three: Issues at the Individual Practice Level

#### Ideas for Interventions

##### Continuing Professional Development

Educational seminars could help teach about coping with stress, as well as a wide range of other educational and practice needs elaborated as follows:

Make available courses (not mandatory) re: emotional problem management.

Dealing with death or mistakes.

Need help & advice on how to avoid lawsuits........

Provide some personnel management courses, i.e. how to tell if an office assistant is going to be useful prior to hiring them.

Educational seminars provide insight on how to strengthen the physician-patient relationship and physicians indicate what areas are needed:

Understand the advocacy stances of interpreters.

Improving communication and empathy towards patients especially those of different cultures.

How to deal with adverse/violent patient and families.

Cultural teaching sessions.

##### Diversifying Medical Practice

Applying a range of practice solutions helps to prevent practice stagnation, and increase personal accomplishment. This can be facilitated by experiential learning and the following are suggestions provided by physicians:

I am of an ethnic minority and speak another language, this helps me understand ethnic groups. Have worked in foreign countries 21 different times, 3–7 weeks at a time, so I am familiar with all kinds of cultural variations and expectations and build them into my attitude in dealing with different cultural and socioeconomic backgrounds.

Blend work life. I do part time clinical practice, health research & health administration. The mix provides a good balance in achieving short, medium & long-term goals, respectively. I work at three different sites.

##### Health Policy Initiatives

These are initiatives that help to improve access, quality of care, cost of care, and have a major impact on the delivery of care. Physicians understand the impact that lack of policy has on their practice, and recommend that policies be formulated in the following areas:

More established and experienced bank of interpreters.

Increase support for office based practice (management training, management tools).

Encourage and help fund more CME.

Reinstate clinical practice guidelines so individuals do not have to keep up with the literature in all areas- can depend on guidelines in some practice areas.

Advocate for physician work hour reform. Advocate for streamlining billing & licensing procedures.

### Theme Four: Work/Life Balance (Micro level)

#### What causes imbalance

##### Family Responsibilities

Responsibility in relation to one's spouse, partner, dependents, children or other family members to whom one is liable for care and support. Work/life conflict is now a pressing concern for health care professionals, and families in general, and are expressed as follows:

Pressures of home life/family competing with work pressures.

Child and elderly parent issues (illness, disabilities, school).

Demands of caring for 4 young children while husband has own medical career..............

Being a solo parent, sometimes hard to sympathize with others not carrying this combined workload.

Parental health concerns, family conflict.

Worry regarding nuclear family development and extended family challenges.

##### Physician Unhealthiness

These are symptoms or behaviours that contribute to or cause poor mental or physical health described as:

My own physical health is fragile so balancing my need for rest with my responsibilities requires savvy.

Chronic headaches/migraines.

Lack of sleep due to infant children.

Not enough energy to care for self. i.e. exercise.

Major depression in 92.

##### Heavy Workload

High levels of physical or psychologically intense work over prolonged periods taxes a person's ability to meet the demands in their personal and professional life, leading to emotional exhaustion. This is a major cause of burnout and is described as follows:

It is unclear why in 2006 some/most residents still are in the hospital for over 100 hours some weeks and we are looking for reasons why people may be experiencing stress, job dissatisfaction and feelings of not being in control of ones life.

Not being able to take time off work because of own medical illness – minor like colds, GI infections, because no one to provide back-up.

Being on call – hours are unhealthy and demoralizing.

##### Constraints on Personal Time

A heavy workload with an irregular and unpredictable work schedule and sometimes-unsupportive community creates an imbalance between work and personal time and are described as follows:

Being away from my family and not being accepted in Canada as a foreigner.

Lack of time to cultivate friendships out of work place.

Dealing with some contentious issues in my own community (outside my work).

No quality time to spend with wife and kids.

##### Unpredictability in Work and Home Life

Careful planning at home does not always ensure that things turn out as we wish, causing anxiety and stress when they don't. The difficulties associated with being on call and staffing problems, create difficulties fulfilling obligations in one's personal life:

Vacation time schedule being pulled or change of schedules.

Unpredictable events in family life.

##### Financial Concerns

Unexpected costs require adjustments to other financial obligations, which create a re-alignment between wants and needs:

Moderate financial concerns i.e. children in university out of town, requiring support.

### Theme Four: Work/Life Balance

#### Ways to Restore

##### Health Education

These are learning experiences which favourably influence understanding, attitudes, and behaviour relating to individual as well as community health. The focus is on attitudes, behaviours, and assistance in making informed decisions regarding prevention, behaviour change, and problem solving. Health education needs are described as follows:

Publications/workshops on physician health.

More educational services re: lifestyle etc. More open discussions regarding above issues.

Workshops for physicians to learn of the signs of "burnout" etc.

Time and life management.

Teaching sessions regarding balanced lifestyle for physicians.

Perhaps Physician Wellness Centre or Cont Ed could organize 1/2 day or 1–2 day workshops on coping/communications/stress. However, for privacy, I would prefer to do it in another province.

Programs, sessions about managing stress.

Workshops in self-care.

##### Health Promotion

Health promotion is a process of enabling people to increase control over and improve their health, and is both a philosophy (way of being) and a practice (way of doing). This change is facilitated through increased awareness, behavioural change, and emphasis on creating environments that support good health practices. To promote physician health, the following are recommended:

Increase availability or provide incentive/facilitation to allow for personal mental/physical health initiatives re: reduced gym membership, etc.

Promote time for self e.g. exercise, massage, other stress reduction, health promotion activities. Hire a massage therapist for their members.

Liaise with businesses to offer recreational activity, deals, packages, but could be less costly if the numbers are right.

Need physician health team to help physicians find their own family doctors etc.

Active role in med school, residency + beyond to give physicians practical advice on ways to lower stress, make positive changes in work environment etc.

##### Community and Social Outlet

Having a social outlet provides a sense of social connection and a way to express oneself in the surrounding environment. Included are relationships at work, with family, friends and community groups:

Identify solo specialists who are at risk by being isolated/encourage members to have an active family life/set boundaries at work particularly encourage members to talk regularly with their colleagues about their patients and their personal issues. This really helps.

I am less stressed at this particular point in my life than any other period. I only practice within my cultural group for the most part. Have never required an interpreter.

##### Healthy Work Place Initiatives

These are initiatives that improve the physical environment (safety, ergonomics) and organisational climate (supportive culture, autonomy), and promote wellbeing:

Reduce on call shifts for residents. No one should ever be expected to do in-house call for 24–30 hrs straight!

Better maternity leave benefits. Help with after hours childcare when on call.

Make it easier again to "import" doctors from outside Canada to lighten the workload.

By finally establishing our fee structure to pay us for things like committee work and after hrs premiums, I am now able to work less office hours and maintain my income. This means more free time and a much happier and effective & efficient doc.

Provide back-up services and locums.

Continue supporting initiatives such as parental leave and reduced fees for part-time working members.

Look for ways to make practice more attractive so the number of physicians would increase and to make the workload decrease.

## Discussion

### Governance and Health Policy

The complaint and disciplinary process should be transparent, fair to physicians and improve patient outcomes. Research pinpointing the causes and outcomes of complaints, should be made available and used to develop seminars to inform and educate physicians. The introduction of stress management courses in one study reduced the rate of malpractice claims from 31 to 9 after the intervention, and medication errors fell from a monthly mean of 10.3 to 5.1 after the intervention. [8] Improving communication skills training and professional development workshops with emphasis on the explanation phase of the medical interview should also be initiated. [15] The use of an administrative physician to deal directly with patients and family members, who have contentious behaviours and insistent demands, is also suggested as having potential to lower avoidance behaviours of primary care physicians. [39] Opportunities to review research findings, to practice communication skills in realistic situations, speaking openly with patients, families and colleagues, all have potential to reduce the incidence and effects of complaints. [27,39]

Doctors are now being asked to consider the effectiveness and cost of care as part of patient management, mindful of protocols and guidelines.[31] Research derived from the practice environment to identify the gaps between the actual practice environment, and institutional management directives can facilitate joint working arrangements, collegiality, and facilitate clinical leadership. [18,31]

### Health Human Resources and Education

Across Canada, health care settings are facing ever-increasing staff shortages. Effective physician staffing requires extraordinary efforts in recruitment and retention. [6] Retaining physicians in rural and remote regions remains a challenge, and in particular, professional and lifestyle issues are most prominent. [6] There is also a growing demand for flexibility in medical practice, for all physicians especially young, female, and those focused on healthy ageing. Innovative strategies such as flexible and family-friendly working practises, personal development, educational opportunities, job enrichment, and appropriate working hours can enhance motivation and improve performance. [6] Research suggests that human resource planners should anticipate decreases in physician full-time equivalents due to the greater numbers of women in medicine who desire to work fewer hours. [30] The issue of work-life balance touches upon nearly every aspect of health human resources, in terms of how health care organisations will develop and implement policies that foster a healthy equilibrium between the professional and personal lives of physicians.

## Conclusion

Our mixed method approach has shed light on understanding physician wellbeing. Our qualitative findings reflect prior research findings from Australia, United States, and Europe, despite differences in health systems, rules and policies. We also demonstrated that the socio-ecological model is a useful framework to analyze wellbeing with its systems approach, which relates to issues at the structural, organisational, and individual levels. No existing studies or literature in Canada could be found examining wellbeing at the service delivery level, although the responses we obtained from the Manitoban physicians suggests that findings and conclusions from other studies at organisational levels may be applicable to the Canadian context as well. [9,10] In the Canadian health care system regional health authorities have responsibility for health service delivery, the College of Physicians and Surgeons has responsibility for licensing and complaint process, and the medical associations negotiate with provincial governments for salaries and fee for service. This requires coordinated efforts across many levels to develop the appropriate policies and programs, as stressors and interventions have been identified by the physicians with application to all of these organisations.

We also determined from our separately presented quantitative study, that work -life conflict, lack of autonomy, and emotional exhaustion were associated with physical symptoms of stress. [33,40] Females reported higher levels of physical symptoms of stress than males, and used a greater range and frequency of coping mechanisms. [40] Similar findings from Norway also showed female physicians with greater health complaints than males. [4] Subjective health complaints were also reported as a result of job dissatisfaction and emotional exhaustion. [4] We also cross-compared reported physical symptoms of stress from our quantitative study to reported symptoms of stress from our qualitative study in the work-life balance theme, which received the highest number of responses from the physicians. The second highest number of responses was the external constraints on the practice of medicine theme, where issues such as lack of autonomy and resources contribute to stress, poor health, and dissatisfaction.

A weakness of our qualitative result was that we were unable to expand upon the written responses because we did not conduct interviews allowing for follow-up questions. Secondly some of the responses and comments made were short, and could have benefited from more detail. However, we received a great variety of answers allowing us to reveal multiple factors related to physician health and wellbeing.

Future research should target interventions at the organisational and institutional levels that have potential to decrease stress levels and increase career satisfaction. Professional development programs, and human resource policies, are especially needed to address work-life conflict and job dissatisfaction. Systemic health care difficulties remain a challenge especially due to lack of resources, heavy workload, and administration. Training in stress management, and healthy coping mechanisms should be explored for their potential to improve job satisfaction and to promote greater life balance.

## Competing interests

The authors declare that they have no competing interests.

## Authors' contributions

BL participated in the design of the study, collected the data, analyzed and interpreted the data, drafted the abstract and conference presentation in PowerPoint, wrote the initial and revised manuscripts. RL participated in the design of the study, analyzed and interpreted the data, critically evaluated the abstract and conference presentation in PowerPoint, critically evaluated the manuscript draft and final manuscript for important intellectual content, approved the final version for publishing. EF analyzed and interpreted the data, critically evaluated the abstract and conference presentation in PowerPoint, presented the manuscript at an international conference, critically evaluated the manuscript draft and final manuscript for important intellectual content, approved the final version for publishing. All authors read and approved the final manuscript.

## Pre-publication history

The pre-publication history for this paper can be accessed here:



## References

[B1] World Health Organisation (1998). Geneva: Health promotion glossary. http://www.who.int/healthpromotion/about/HPG/en/.

[B2] Frank E (2004). Physician health and patient care. JAMA.

[B3] Frank E, Biola H, Burnett CA (2000). Mortality rates and causes among U.S. Physicians. Am J Prev Med.

[B4] Aasland OG, Olff M, Falkum E, Schweder T, Ursin H (1997). Health complaints and job stress in Norwegian physicians: The use of an overlapping questionnaire design. Soc Sci Med.

[B5] Arnetz BB (2001). Psychosocial challenges facing physicians of today. Soc Sci Med.

[B6] Dubois CA, McKee M, Rechel B, Rechel B, Dubois CA, McKee M (2006). Introduction: Critical challenges facing the health care workforce in Europe. The Health Care Workforce in Europe.

[B7] Chan AO, Huak CY (2004). Influence of work environment on emotional health in a health care setting. Occup Med (Lond).

[B8] Firth-Cozens J (2001). Interventions to improve physicians' wellbeing and patient care. Soc Sci Med.

[B9] Freeborn DK (2001). Satisfaction, commitment, and psychological wellbeing among HMO physicians. West J Med.

[B10] Jansson von Vultee P, Axelsson R, Arnetz B (2007). The impact of organisational settings on physician wellbeing. Int J Healthcare Qual Assur.

[B11] Creswell JW, Clark VP (2007). Designing and Conducting Mixed Methods Research.

[B12] Onwuegbuzie AJ, Leech NL (2006). Linking research questions to mixed methods data analysis procedures. TQR.

[B13] Dubé L, Teng L, Hawkins J, Kaplow M, Savage GT, Blair JD, Fottler MD (2002). Emotions, the neglected side of patient-centered health care management: The case of emergency department patients waiting to see a physician. Advances in Health Care Management.

[B14] Kristiansen IS, Forde OH, Aasland OG, Hotvedt R, Johnsen R, Forde R (2001). Threats from patients and their effects on medical decision making: a cross-sectional, randomized trial. Lancet.

[B15] Anderson K, Allan D, Finucane P (2001). A 30-month study of patient complaints at a major Australian hospital. J Qual Clin Pract.

[B16] Lammers JC, Duggan A (2002). Bringing the physician back in: Communication predictors of physicians' satisfaction with managed care. Health Commun.

[B17] Veldhuis M (1994). Defensive behaviour of Dutch family physicians. Widening the concept. Fam Med.

[B18] Davis D, Barnes B, Fox R (2003). The Continuing Professional Development of Physicians: From Research to Practice.

[B19] Matern U, Koneczny S (2001). Safety, hazards and ergonomics in the operating room. Surg Endosc.

[B20] Hall P, Keely E, Dojeiji S, Byszewski A, Marks M (2004). Communication skills, cultural challenges and individual support: Challenges of international medical graduates in a Canadian healthcare environment. Med Teach.

[B21] O'Neale Roach J, Guthrie E (2000). Dealing with stress: Medical schools should teach us how to cope. student BMJ.

[B22] Frank E, Carrera JS, Stratton T, Bickel J, Nora LM (2006). Experiences of belittlement and harassment and their correlates among medical students in the United States. BMJ.

[B23] Cohen JS, Patten S (2005). Wellbeing in residency training: A survey examining resident physician satisfaction both within and outside of residency training and mental health in Alberta. BMC Med Educ.

[B24] Cheema S, Ahmed K, Naqvi SA, Giri SK, Kaliaperumal VK (2005). Bullying of junior doctors prevails in Irish health system: A bitter reality. Ir Med J.

[B25] Frank E, Brogan D, Schiffman M (1998). Prevalence and correlates of harassment among U.S. women physicians. Arch Intern Med.

[B26] Campbell S, Delva D (2003). Physician do not heal thyself: Survey of personal health practices among medical residents. Can Fam Physician.

[B27] Aasland OG, Forde R (2005). Impact of feeling responsible for adverse events on doctors' personal and professional lives: the importance of being open to criticism from colleagues. Qual Saf Health Care.

[B28] Values and work of the Finnish Medical Association. http://www.laakariliitto.fi/e/ethics/values.html.

[B29] Lomas J, Woods J, Veenstra G (1997). Devolving authority for health care in Canada's provinces: 1. An introduction to the issues. CMAJ.

[B30] Frank E, McMurray JE, Linzer M, Elon L (2000). Career satisfaction of U.S. women physicians: Results from the women physicians' health study. Arch Intern Med.

[B31] Garelick A, Fagin L (2005). The doctor-manager relationship. Advan Psychiatr Treat.

[B32] Cunningham W (2004). The immediate and long-term impact on New Zealand doctors who receive patient complaints. N Z Med J.

[B33] Lee RT, Lovell BL, Brotheridge CM Tenderness and Steadiness: Relating Job and interpersonal demands and resources with burnout and physical symptoms of stress among Canadian physicians. J Appl Soc Psychol.

[B34] Shanafelt TD, Novonty P, Johnson ME, Zhao X, Steensma DP, Lacy MQ (2005). The wellbeing and person wellness promotion strategies of medical oncologists in the North Central Cancer Treatment Group. Oncology.

[B35] Sammut MR (2006). Family doctors and health promotion: Do we practise what we preach?. Malta Medical Journal.

[B36] Greene JC, Caracelli VJ, Graham WF (1989). Toward a Conceptual Framework for Mixed-Method Evaluation Designs. Educ Eval Policy An.

[B37] Bronfenbrenner U (1979). The Ecology of Human Development.

[B38] Thomas DR (2006). A general inductive approach for analyzing qualitative evaluation data. Am J Eval.

[B39] Gordon GH, Baker L, Levinson W (1995). Physician-patient communication in managed care. West J Med.

[B40] Lovell BL, Lee RT, Brotheridge CM Gender differences in the application of communication skills, emotional labour, stress-coping and wellbeing among physicians: It's in the delivery. Int J Med.

